# Enhanced synergistic antitumor effect of a DNA vaccine with anticancer cytokine, MDA-7/IL-24, and immune checkpoint blockade

**DOI:** 10.1186/s12985-022-01842-x

**Published:** 2022-06-25

**Authors:** Seyed Mohammad Miri, Behzad Pourhossein, Seyed Younes Hosseini, Mohsen Keshavarz, Shohreh Shahmahmoodi, Mohammad Reza Zolfaghari, Seyed Reza Mohebbi, Ali Gorji, Amir Ghaemi

**Affiliations:** 1 Department of Influenza and Other Respiratory Viruses, Pasteur Institute of Iran, P.O. Box: 1316943551, Tehran, Iran; 2 Department of Medical Virology, Hamadan University of Medical Sciences, Hamedan, Iran; 3 Department of Virology, School of Public Health, Tehran University of Medical Sciences, Tehran, Iran; 4 Department of Bacteriology and Virology, Shiraz University of Medical Sciences, Shiraz, Iran; 5 The Persian Gulf Tropical Medicine Research Center, The Persian Gulf Biomedical Sciences Research Institute, Bushehr University of Medical Sciences, Bushehr, Iran; 6 Department of Microbiology, Qom Branch, Islamic Azad University, Qom, Iran; 7 Gastroenterology and Liver Diseases Research Center, Research Institute for Gastroenterology and Liver Diseases, Shahid Beheshti University of Medical Sciences, Tehran, Iran; 8 Department of Neurosurgery and Department of Neurology, Westfälische Wilhelms-Universität, Münster, Germany; 9 Shefa Neuroscience Research Center, Khatam Alanbia Hospital, Tehran, Iran

**Keywords:** MDA-7/IL-24, IL-10 blockade, DNA vaccine, Regulatory T cells, TRAIL, Human papillomavirus

## Abstract

**Background:**

MDA-7/IL-24 cytokine has shown potent antitumor properties in various types of cancer without exerting any significant toxicity on healthy cells. It has also been proved to encompass pro-immune Th1 cytokine-like behavior. Several E7 DNA vaccines have developed against human papillomavirus (HPV)-related cervical cancer. However, the restricted immunogenicity has limited their clinical applications individually. To address this deficiency, we investigated whether combining the E7 DNA vaccine with MDA-7/IL-24 as an adjuvant would elicit efficient antitumor responses in tumor-bearing mouse models. Next, we evaluated how suppression of immunosuppressive IL-10 cytokine would enhance the outcome of our candidate adjuvant vaccine.

**Methods:**

For this purpose, tumor-bearing mice received either E7 DNA vaccine, MDA-7/IL-24 cytokine or combination of E7 vaccine with MDA-7/IL-24 adjuvant one week after tumor challenge and boosted two times with one-week interval. IL-10 blockade was performed by injection of anti-IL-10 mAb before each immunization. One week after the last immunization, mice were sacrificed and the treatment efficacy was evaluated through immunological and immunohistochemical analysis. Moreover, the condition of tumors was monitored every two days for six weeks intervals from week 2 on, and the tumor volume was measured and compared within different groups.

**Results:**

A highly significant synergistic relationship was observed between the E7 DNA vaccine and the MDA-7/IL-24 cytokine against HPV-16+ cervical cancer models. An increase in proliferation of lymphocytes, cytotoxicity of CD8+ T cells, the level of Th1 cytokines (IFN-γ, TNF-α) and IL-4, the level of apoptotic markers (TRAIL and caspase-9), and a decrease in the level of immunosuppressive IL-10 cytokine, together with the control of tumor growth and the induction of tumor regression, all prove the efficacy of adjuvant E7&IL-24 vaccine when compared to their individual administration. Surprisingly, vaccination with the DNA E7&IL-24 significantly reduced the population of Regulatory T cells (Treg) in the spleen of immunized mice compared to sole administration and control groups. Moreover, IL-10 blockade enhanced the effect of the co-administration by eliciting higher levels of IFN-γ and caspase-9, reducing Il-10 secretion and provoking the regression of tumor size.

**Conclusion:**

The synergy between the E7 DNA vaccine and MDA-7/IL-24 suggests that DNA vaccines’ low immunogenicity can be effectively addressed by coupling them with an immunoregulatory agent. Moreover, IL-10 blockade can be considered a complementary treatment to improve the outcome of conventional or novel cancer therapies.

**Supplementary Information:**

The online version contains supplementary material available at 10.1186/s12985-022-01842-x.

## Introduction

Immunization with DNA vaccines, which express a foreign antigen, represents a simple and effective method of generating antigen-specific cellular and humoral immunity in various animal models [1, 2]. Due to the cellular uptake and increased expression of antigens within host cells, DNA vaccines can stimulate specific immune responses in mice models while causing no pathogenic infection in vivo [3, 4]. However, DNA vaccines are not widely used because of their limited immunogenicity, especially in large animals and clinical trials [5]. Therefore, several approaches have attempted to promote the immunogenicity and efficacy of DNA vaccines, particularly the incorporation of genetically encoded immune adjuvants aimed at enhancing the immunogenicity of antigens [6].

The administration of genetic adjuvants not only augments the quantity but also modulates the type of immune responses to DNA vaccines [7]. Such adjuvants comprise plasmid-encoded immunomodulatory molecules, including cytokines, chemokines, immune stimulatory molecules such as toll-like receptor (TLR), agonists and interferon (IFN) regulatory factors, or inhibitors of immune suppressive pathways [2, 8].

After discovering melanoma differentiation-associated gene-7 (MDA-7) in 1993, the cytokine-like behavior of MDA-7 along with the existence of an IL-10 family signature among the MDA-7 gene sequence encouraged the Human Gene Organization to entitle it IL-24, which together constitute its name, the MDA-7/IL-24 [9]. The MDA-7/IL-24 has shown promising therapeutic anticancer activity in clinical trials [10, 11]. There are a broad range of mechanisms through which MDA-7/IL-24 acts as a powerful anti-cancer agent, including the inducement of apoptosis and autophagy, the regulation of angiogenesis, invasion and metastasis, the propagation of anti-cancer responses to nearby malignant cells through bystander effect, as well as the enhancement of anti-cancer function of other therapeutic methods as a synergistic agent [12].

Recently, MDA-7/IL-24 has been reported to augment the frequency of IFN-γ-expressing CD8+ T cells and influence the development of antitumor immune responses, suggesting MDA-7/IL-24 as a pro-Th1 cytokine [13]. However, the role of MDA-7/IL-24 in the stimulation of vaccine-induced antigen-specific T cell responses remains unclear. Therefore, in the current study, we evaluated whether MDA-7/IL-24 could be a candidate vaccine adjuvant to induce antigen-specific cellular immunity and enhance antitumor immune responses in a mouse model of the HPV-associated tumor when combined with an E7 DNA vaccine.

Furthermore, owing to the role of IL-10 cytokine in the progression of HPV-positive cervical cancer cells [14], we assessed if the IL-10 blockade improved the efficacy of the E7&IL-24 candidate vaccine against TC-1 tumor cells.

## Materials and Methods

### Cell lines and animals

The murine tumor cell line, TC-1, part of the Johns Hopkins Special Collection, derived from primary epithelial cells of C57BL/6 mice, co-transformed with HPV16 E6 and E7, and activated c-Ha-ras oncogene, then used to induce tumor formation within studied groups (H-2^b^ murine system).

The EL4 cells, a mouse lymphoma cell line derived from C57BL/6-Ly5.2 mice, and human embryonic kidney cells (HEK-293T) were obtained from the National Cell Bank of Iran (NCBI, Pasteur Institute, Tehran). In this study, The EL4 cell line, presenting E7 peptide, was employed as the target in the cytotoxicity assay (LDH assay). HEK-293T cell line was used to confirm the recombinant genetic adjuvant expression. In the first step, the cell lines were cultured in RPMI 1640 (Gibco, Thermo Fisher Scientific, USA) supplemented with 10% heat-inactivated fetal bovine serum (FBS) (Gibco, Thermo Fisher Scientific, USA), insulin, growth factor, 2 mM L-glutamine, 1 mM pyruvate, 0.1 mM nonessential amino acids, 100 units penicillin and 100 μg Streptomycin. Female 6-to-8-week-old C57BL/6 mice were purchased from the Pasteur Institute of Iran. They were maintained on a 12-h light/dark cycle at a room temperature between 20 and 22 °C and had free access to food and water. All animal manipulations were carried out according to the Ethical Committee for the use and care of laboratory animals of Pasteur Institute of Iran (ethics number: et-1137).

### Preparation of recombinant expression plasmids

Recombinant plasmid constructs were verified by DNA sequencing and gene expression. Briefly, the *E. coli* bacterial strain DH5α competent cells (Pasteur Institute of Iran) were transformed through the CaCl_2_ method and then cultured in Luria–Bertani (LB) medium to amplify plasmids.

Stocks of endotoxin-free DNA vaccine, pcDNA3.1-MDA/IL-24 and vector control plasmids (pcDNA3.1) in 0.1 M PBS were purified for vaccination using the EndoFree Plasmid Giga Kit (Qiagen, Hilden, Germany) with DNA being dissolved in endotoxin-free Tris–EDTA (Sigma, St. Louis, MO). The confirmation of recombinant plasmids was achieved by restriction analysis.

### In vitro and in vivo expression confirmation

The expression of E7 in the DNA vaccine was confirmed in our previous study [1]. Transfection for transient expression of MDA/IL-24 in the Human embryonic kidney-293 cell line (HEK-293) was carried out by a Lipofectamine 2000 reagent according to the manufacturer’s instructions (Thermo Fisher Scientific, USA). The expression of MDA/IL-24 (pcDNA3.1-MDA/IL-24) by transduced HEK293 cells was performed using a monoclonal antibody against MDA/IL-24 (R&D. cat number: MAB2786). As a control, whole-cell lysate of HEK 293T cells transfected with an empty pcDNA3.1expression plasmid was used.

### Experimental tumor model establishment/immunization procedure

The C57BL/6 mice were challenged by subcutaneous (S.C) injection with 7 × 10^5^ TC-1 (ATCC) cells in 100 μL PBS, which constitutively express wild type HPV16 E6/E7. The mice (n = 10) were randomly divided into five groups (10 mice per group). After one week, the mice were immunized with different formulations.

Two groups were intratumorally administered with either 90 μg of the plasmid encoding HPV-16 E7 (DNA vaccine) or the plasmid expressing MDA/IL-24 (pcDNA3.1-MDA/IL-24, control adjuvant) three times at seven-day intervals. The same immunization schedule was applied to the adjuvanted vaccine group (E7&IL-24) using the DNA vaccine encoding HPV-16 E7 (90 μg) in combination with 90 μg of the plasmid expressing MDA/IL-24. The pcDNA3.1 and phosphate buffer saline (PBS) were used as control groups.

**Fig. 1:**
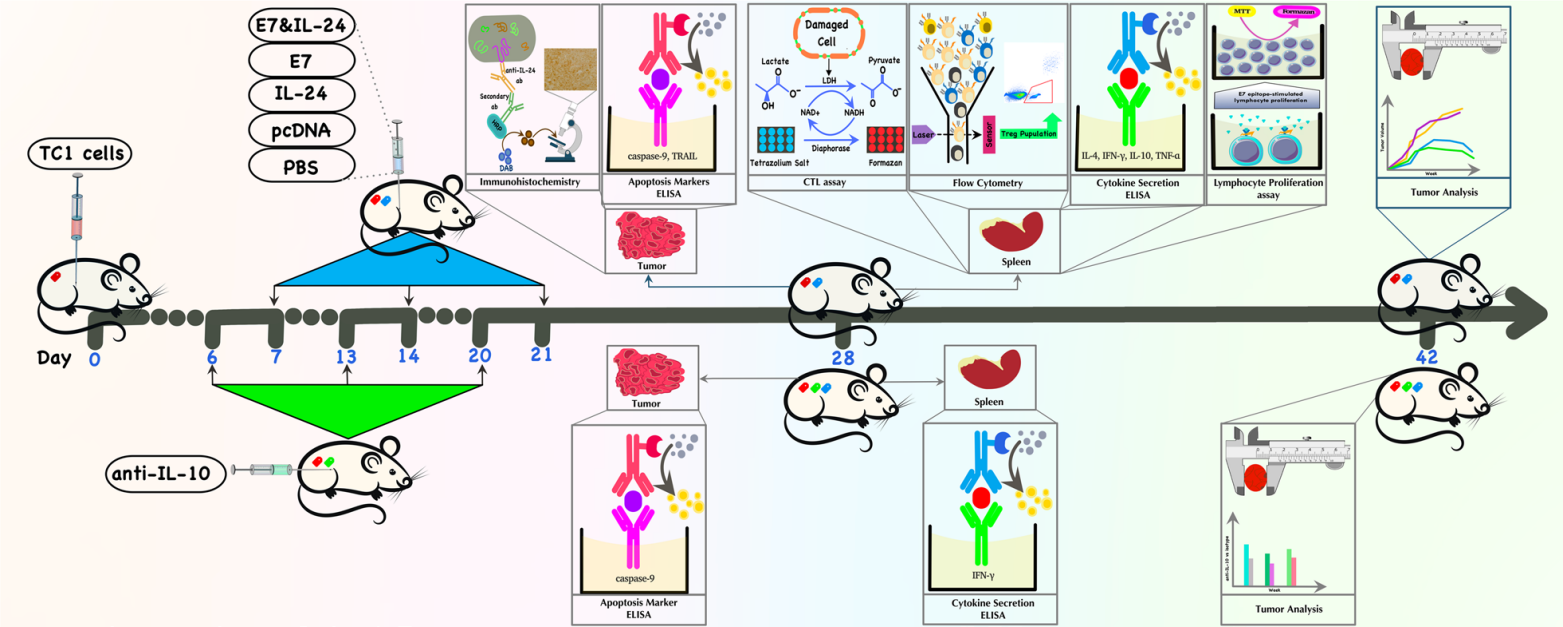
Schematic overview of all analyses from day 0 to day 42. Briefly, 1 week following tumor challenge (day 0), mice were immunized with E7, IL-24, or E7&IL-24; or received control reagents (pcDNA or PBS). This procedure was repeated 2 times at one-week interval. One week later (day 28), experiments were conducted regarding lymphocyte proliferation, cytokine secretion (using ELISA), CTL responses, caspase-9 and TRAIL levels (using ELISA), Treg population (using flow cytometry) and anti-IL-24 immunohistochemistry. Finally, on day 42, the last tumor analysis was performed (following every 2 day monitoring routine from day 14), and data interpretation was applied. In parallel (as depicted under the timeline), a group of mice received anti-IL-10 one day before each immunization. On day 28, the level of IFN-γ, as well as caspase-9, was measured. On day 42, the final tumor data were obtained and compared with the results of isotype control groups. Description of label colors on mice bodies: red = tumor-bearing mouse; blue = immunized mouse; green = anti-IL-10-received mouse

The tumor size was measured by tissue calipers every two days and the tumor volume was calculated using the Carlsson formula [15]. The Mean tumor volume (Y-axis) was plotted to show in vivo growth curves (seven mice per group) using the formula: volume = (width)^2^ × length/2. The tumor growth was examined for at least forty-two days. The different aspects of immunity (lymphocyte proliferation (MTT), cytotoxicity (LDH), cytokine levels (ELISA), and regulatory T cell population (flow cytometry)) were studied one week after the third administration. Results are representative of three independent experiments (three mice per group). The statistical analysis was done using one-way ANOVA. The final data represent the mean ± standard deviation (S.D.) of three measurements. The schematic overview of all experimental procedures has been depicted in Fig. 1.

### Lymphocyte proliferation assay (LPA)

One week after the last vaccination, lymphocyte proliferation of splenocytes was measured (three mice/group). The spleens harvested from individual mice were minced and passed through a nylon sieve, and red blood cells were lysed using red blood cell lysis buffer containing 0.75% NH_4_Cl in Tris-buffer. Single-cell suspensions from mice in each group were prepared under sterile conditions. The cell number and viability were determined by the trypan blue exclusion test [3]. Splenocytes at a concentration of 2 × 10^5^ cells/well were propagated in the 96 well plates in the presence of 1 μg of synthetic E749–57-specific H-2Db CTL epitope (biomatik, Canada) or in the absence of stimuli (medium only).

Evaluation of the lymphocyte proliferation was performed using the MTT (3-(4,5-dimethylthiazol-2-yl)-2,5-diphenyltetrazolium bromide) kit (Sigma), based on colorimetric reaction. After 72 h of incubation at 37 °C and 5% CO_2_, 10 μg/μl of the MTT solution was added to each well. Then, they were incubated under the same condition for five hours. At the end of the incubation period, the supernatant was removed, and 100 μl of the solution buffer was added to the wells to make purple soluble formazan crystals. The plate was read using an ELISA reader (BIOTEK) at 540 nm and the OD was recorded to calculate the stimulation index (SI). The SI was determined by the formula: SI = (Cs ^_^ Cu) ∕ Cu, in which Cs is the OD of stimulated cells and Cu stands for relative cell numbers of unstimulated cells.

### Cytotoxic T Lymphocyte (CTL) assay

CTL activity was analyzed by lactate dehydrogenase (LDH) release assay [15]. One week after the last administration, single-cell suspensions of splenocytes were prepared and used as effector cells. EL4 cells (as target cells stimulated with 1 μg/μl of the synthetic E7 specific epitope (H-2Db CTL epitope)) in a volume of 100 μl were incuted with effector cells (100 μl) at different effector/target ratios. The release of LDH upon cell lysis was measured with an LDH release assay kit (Takara) according to the manufacturer’s instructions. For low and high controls (spontaneous release and maximum release, respectively), 100 μl of assay medium or 2% Triton X-100 was added. The percentage of specific cytolysis was calculated by the formula:

$${\text{\% }}\,{\text{Cytotoxicity}} = \frac{{\left( {{\text{experimental}}\,{\text{value}} - {\text{effector}}\,{\text{cell}}\,{\text{control}}} \right) - {\text{low}}\,{\text{control}}}}{{{\text{high}}\,{\text{control - low}}\,{\text{control}}}} \times 100.$$

### Cytokine secretion assay

One week after the last immunization, splenocytes of immunized mice were cultured in 24-well plates for three days in phenol red-free RPMI 1640 supplemented with 10% FBS, 2 mM L-glutamine, 25 mM HEPES and 0.1% penicillin/streptomycin, and pulsed with 1 μg/ml E7-specific H-2Db CTL epitope at 37 °C in 5% CO_2_. The supernatants were assayed for the presence of IFN-γ, IL-4, IL-10, and TNF-α using commercially available sandwich-based ELISA kits (R&D systems, San Diego, USA) following the manufacturer’s instruction.

### Intratumoral caspase-9 and TRAIL activity

In order to understand the role of intrinsic apoptosis in anti-tumor responses of the proposed vaccine, the level of caspase-9 and TRAIL in the tumor microenvironment was measured by caspase ELISA kit (Abcam, Cambridge, MA, USA) and Mouse TRAIL ELISA kit (Abcam, Cambridge, MA, USA), respectively. Briefly, one week after the third vaccination, the tumor tissue was extracted from each group (n = 3) and 100 mg of discarded tissue was homogenized in 0.5 ml lysis buffer (0.1 M Tris–HCl pH = 7.6, and 0.1 M fresh dithiothreitol). After centrifugation at 10,000×*g* (1 min), an equal amount of supernatant was added to the substrate‐containing reaction buffer (0.1 M dithiothreitol and 5 μl of 4 mM DEVD-p-NA) and incubated for 120 min at 37 °C. Finally, the caspase-9 and TRAIL activity was assessed by the microplate reader (BioTek, 800TS, USA) at an absorbance of 405 nm. Each experiment was repeated in triplicate.

### Flow cytometric analysis of regulatory T (Treg) cells

To evaluate the Regulatory T (Treg) population in the spleen of immunized mice, the freshly prepared splenocytes were analyzed by flow cytometry using eBioscience Mouse Regulatory T Cell Staining Kit. Briefly, splenocytes (1 × 10^6^/well) were cultured for 5 h in complete RPMI-1640 alone (negative control) or co-cultured with E7-specific H-2Db CTL epitope antigens. The antibodies and reagents used for staining were as follows: FITC-conjugated anti-CD4, APC-conjugated anti-CD25, and internal marker (Foxp3 (PE)). The samples were analyzed using BD FaxCalibur flow cytometry device.

### Immunohistochemical analysis

The transplanted TC-1 tumors were harvested, fixed in a 10% formaldehyde solution, embedded in paraffin, and cut into slices using standard procedures. After antigen retrieval (10 mM sodium citrate buffer, pH = 6.0), endogenous peroxidases were blocked by hydrogen peroxide 3% in PBS for 10 min. Next, tissue sections were treated with the primary anti-IL-24 antibody (R&D systems) diluted at 1:500 concentrations in blocking buffer for 24 h at 4 °C. Afterward, sections were treated with the horseradish peroxidase (HRP)-conjugated secondary antibody at 1:100 dilutions and visualized using DAB plus chromogen substrate (Dako, Agilent, CA, USA) and hematoxylin counterstain. Regarding quantification, five fields of view from at least four separate tissue sections were counted for each group (n = 3) using image J software. The number of positive brown-stained cells over the total number of cells was estimated and used to determine the percentage (%) of staining area.

### IL-10 blockade

In order to evaluate the effect of inhibition of immunosuppressive IL-10 cytokine on the outcome of candidate vaccine and one day prior to each immunization, mice (n = 10) were injected intra-peritoneally (i.p) with 1 mg of either anti-IL-10 receptor (IL-10R) mAb (Biolegend; clone 1B1.3a), which specifically binds the ligand-binding domain of IL-10R, or with IgG1 isotype control mAb (Biolegend). Seven days following the last vaccination (twenty-eight days post tumor challenge), the level of E7-specific IFN-γ and IL-10 in the spleen and the level of caspase-9 in the tumor microenvironment (n = 3) were determined (as described previously). The tumor volume was also monitored up to six weeks after tumor challenge (n = 7) as described in the previous section.

### Statistical analysis

To compare results between the different groups, the one-way univariate analysis of variance (ANOVA) plus multiple comparisons were performed. The statistical software SPSS 11.0 was utilized for statistical analysis. The probability values of *P < 0.05, **P < 0.01, and ***P < 0.001 were considered to demonstrate statistical significance.

## Results

### Expression of MDA-7/IL-24 protein

The expression of MDA-7/IL-24 was verified by western blot with anti-IL-24 (Additional file 1: Fig. S1). The western blot assay proved the presence of IL-24 as a single 24-kD band in the samples in comparison with control vector (pcDNA 3.1 control).

### Lymphocyte proliferation assay (LPA)

**Fig. 2:**
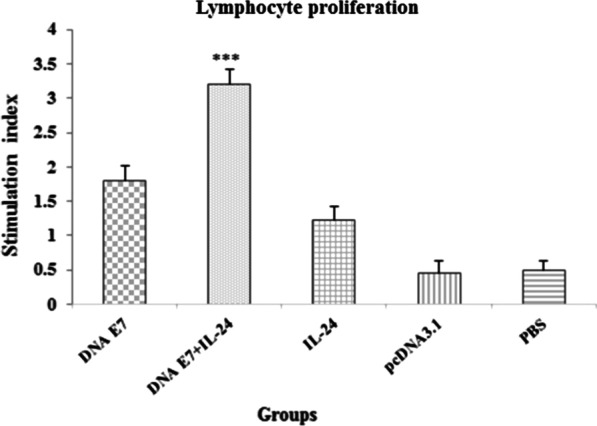
Proliferation of immunized BALB/c mice splenocytes after in vitro re-stimulation with synthetic E7 specific epitope. Splenocytes from mice were harvested one week after the last immunization, and MTT assay evaluated lymphocyte proliferation. Results represent the mean ± SD of 3 mice per groups. ***Statistically significant differences between the E7 DNA plus IL-24 adjuvant compared with other groups (E7 DNA, IL-24, pcDNA, PBS) as determined by one-way ANOVA (P < 0.001)

LPA was used to assess E7-specific T-lymphocyte responses. It was observed that LPA was statistically significant (P < 0.001) in the E7&IL-24 group compared to E7 and IL-24 groups. In addition, although LPA was meaningfully higher (P < 0.001) in all study groups (E7&IL-24, E7, and IL-24,) in comparison with control groups (PBS and pcDNA3), no significant difference was observed between E7 and IL-24 groups (Fig. 2). The findings report a significant synergism between DNA vaccine and genetic adjuvant and the potential of IL-24 as the adjuvant for the induction of cellular immunity.

### Cytotoxicity assay (LDH test)

**Fig. 3:**
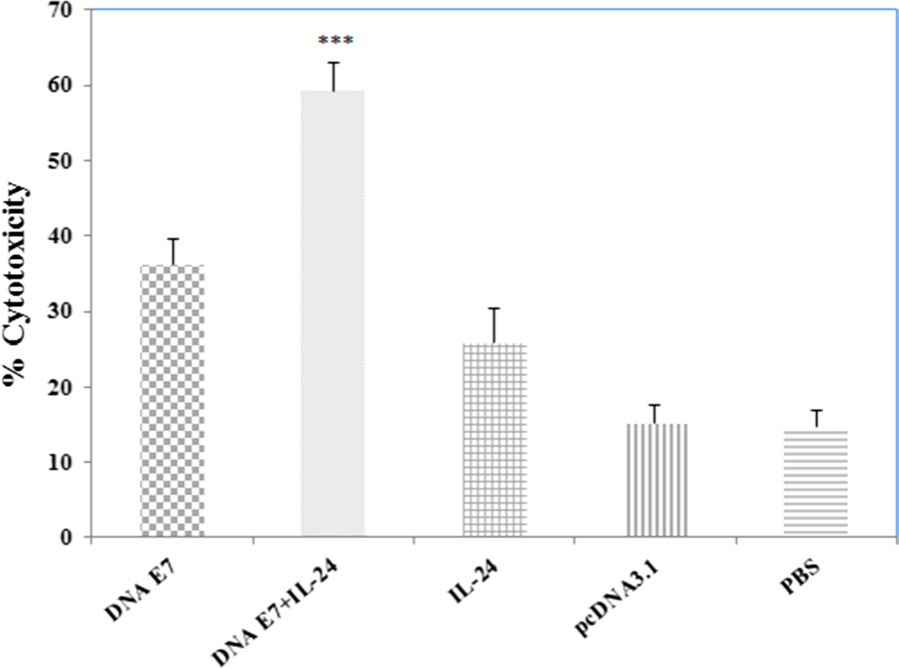
CTL activities of spleen lymphocytes from the tumor-bearing mice were determined by LDH release assay. Splenocytes from immunized mice were isolated one week after last treatment and incubated with EL4 cells; then, pulsed with synthetic E7 specific epitope (H-2Db CTL epitope) at 50:1 effector-(splenocytes)-to-target (EL4) cell (E/T) ratios. Splenocytes derived from immunized mice with DNA E7 plus IL-24 showed higher cytotoxicity against E7 specific epitope re-stimulation than those from the other groups. ***Statistically significant differences between the E7 DNA plus IL-24 compared with other groups (DNA E7, IL-24, pcDNA, PBS) as determined by one-way ANOVA (P < 0.001)

To study whether the IL-24 adjuvant in combination with the DNA vaccine can augment a specific cytotoxic action against transplanted tumors, cytotoxic T lymphocyte responses were evaluated one week after the last immunization. The cytotoxicity response in vaccinated mice was examined using LDH release from the killed cells. Cytotoxic lymphocytes from mice splenocytes were re-stimulated in vitro by applying the antigenic epitope of E7 protein processed by EL4 cells. As shown in Fig. 3, cytotoxic activity was significantly higher (P < 0.001) in the E7&IL-24 group as compared to the E7 and IL24 groups. No significant difference was detected between E7 and IL-24 groups in terms of LDH release; however, there was a significant increase in the cytotoxicity level of all study groups in comparison with the control groups (P < 0.001). It seems that E7&IL-24 co-administration can develop strong specific CTL responses among tumor-bearing mice (Fig. 3).

### Cytokine response

**Fig. 4:**
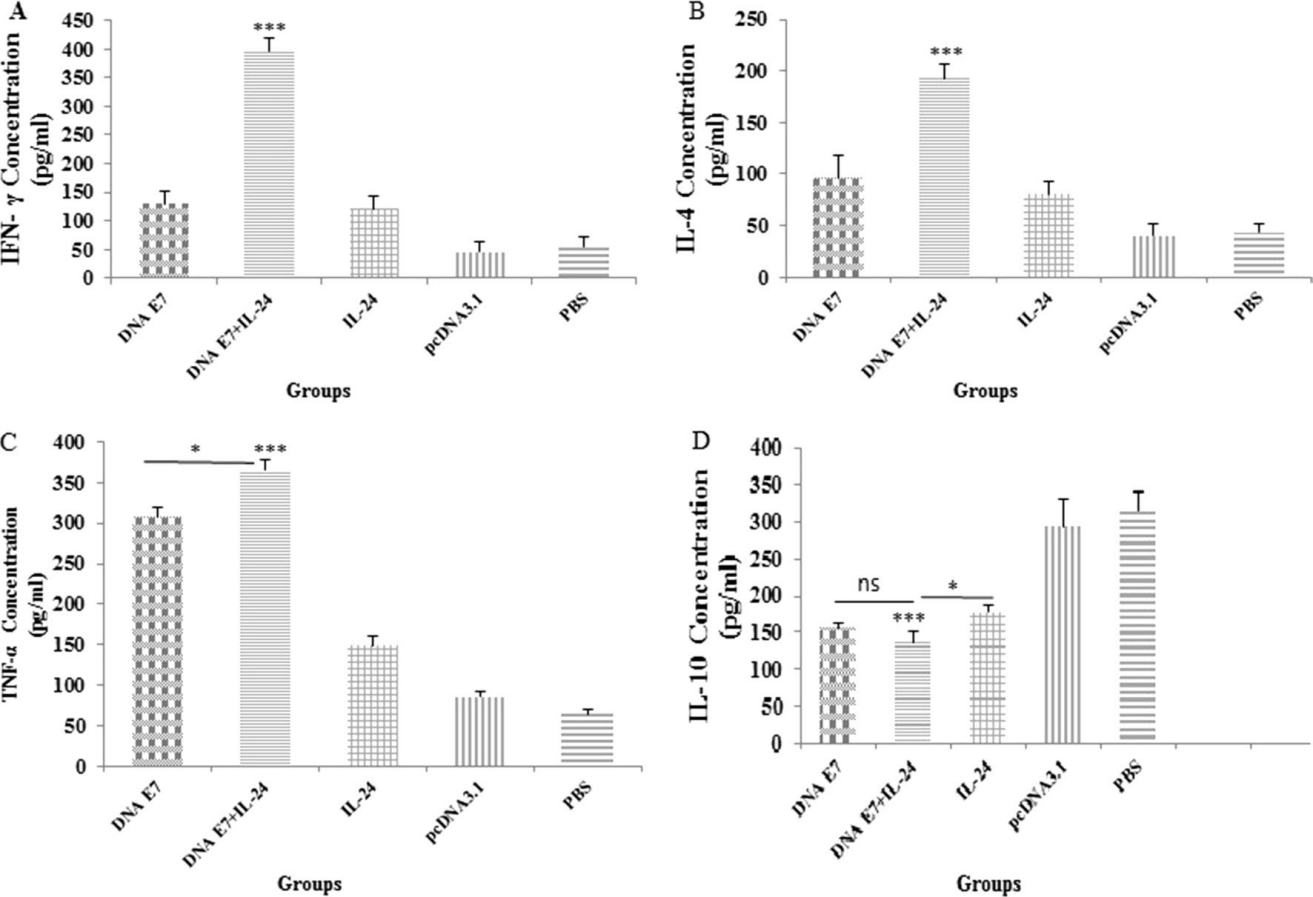
Levels of inflammatory mediators secreted by splenocytes in immunized mice. The supernatants of splenocytes were re-stimulated with E7 specific epitope and collected one week after the last injection. ELISA method was performed to determine the level of IFN-γ (**A**), IL-4 (**B**), TNFα (**C**) and IL-10 (**D**) in splenocyte cultures. Results are representative of three independent experiments and are expressed as the mean ± SD. The DNA E7 adjuvanted with IL-24 induced robust levels of IFN-γ, IL-4 and TNFα when compared to the other groups (**A**–**C**). Also, level of IL-10 (**D**) in DNA E7 adjuvanted with the IL-24 group was significantly reduced compared with other groups (IL-24, pcDNA, PBS). *P < 0.05; **P < 0.01; ***P < 0.001

Cytokine balance was evaluated to determine whether the IL-24 could enhance the antitumor immunity of the adjuvanted DNA vaccine. For the measurement of IFN-γ, IL-4, IL-10, and TNF-α cytokines, the splenocytes of vaccinated mice were co-cultured with EL4 cells, which were primed with E7 antigenic epitope 12 h beforehand. Seventy-two hours after the co-culture, the supernatant was collected and analyzed by ELISA, and the obtained data were assessed using the one-way ANOVA test. As illustrated in Fig. 4A, there was a significant difference (P < 0.001) between the level of IFN-γ in all study and control groups. Interestingly, the simultaneous incorporation of the DNA E7 vaccine and IL-24 noticeably increased (P < 0.001) the IFN-γ level compared to the administration of each component alone. On the other hand, no significant difference was observed between the E7 and IL-24 groups regarding the induction of IFN-γ expression. Overall, it seems that IL-24 can enhance the cell-mediated immunity of our developed E7&IL-24 vaccine probably by promoting the activation of CD8+ T cells and, thereby, the level of IFN-γ.

Based on the data in Fig. 4B, the higher level of IL-4 production was statistically significant (P < 0.001) in the E7&IL-24 group in comparison with E7 or IL-24 groups. While higher levels (P < 0.001) of IL-4 were observed in all study groups rather than in control ones, no significant difference was found in the level of IL-4 between E7 and IL-24 groups. The higher production of IL-4 in the E7&IL-24 group can be a sign of the enhanced proliferation of both helper and cytotoxic lymphocytes, which stimulate humoral and adaptive immunity.

Alpha tumor necrosis factor (TNF-α) cytokine plays an important role in controlling and treating cancers, especially solid tumors. As shown in Fig. 4C, there was a significant difference between the E7&IL-24 group and both E7 (P < 0.05) and IL-24 (P < 0.001) groups concerning the concentration of TNF-α in splenic cells. In addition to the higher levels (P < 0.001) of TNF-α within all immunized groups compared to control ones, a greater amount (P < 0.001) of this cytokine was detected in the E7 group in comparison with the IL-24 group. The higher level of TNF-α in the adjuvant vaccine-administered mice is probably correlated with an increase in cell death processes mediated by this cytokine.

Regarding the production level of IL-10 in study groups and as expected, significantly lower (P < 0.001) amounts of immunosuppressive IL-10 cytokine were found in immunized groups compared with control groups. It is noteworthy that although the difference between the IL-10 production level in E7&IL-24 and E7 groups was not significant, a lower (P < 0.05) level of IL-10 was observed in the E7&IL-24 group when compared to the IL-24 group (Fig. 4D). It seems that IL-24 has a slightly positive impact on controlling the level of IL-10, thus highlighting less detrimental effects of IL-10 on the proliferation of immune cells and the function of antigen-presenting cells.

### Regulatory T cell population analysis by flow cytometry

**Fig. 5:**
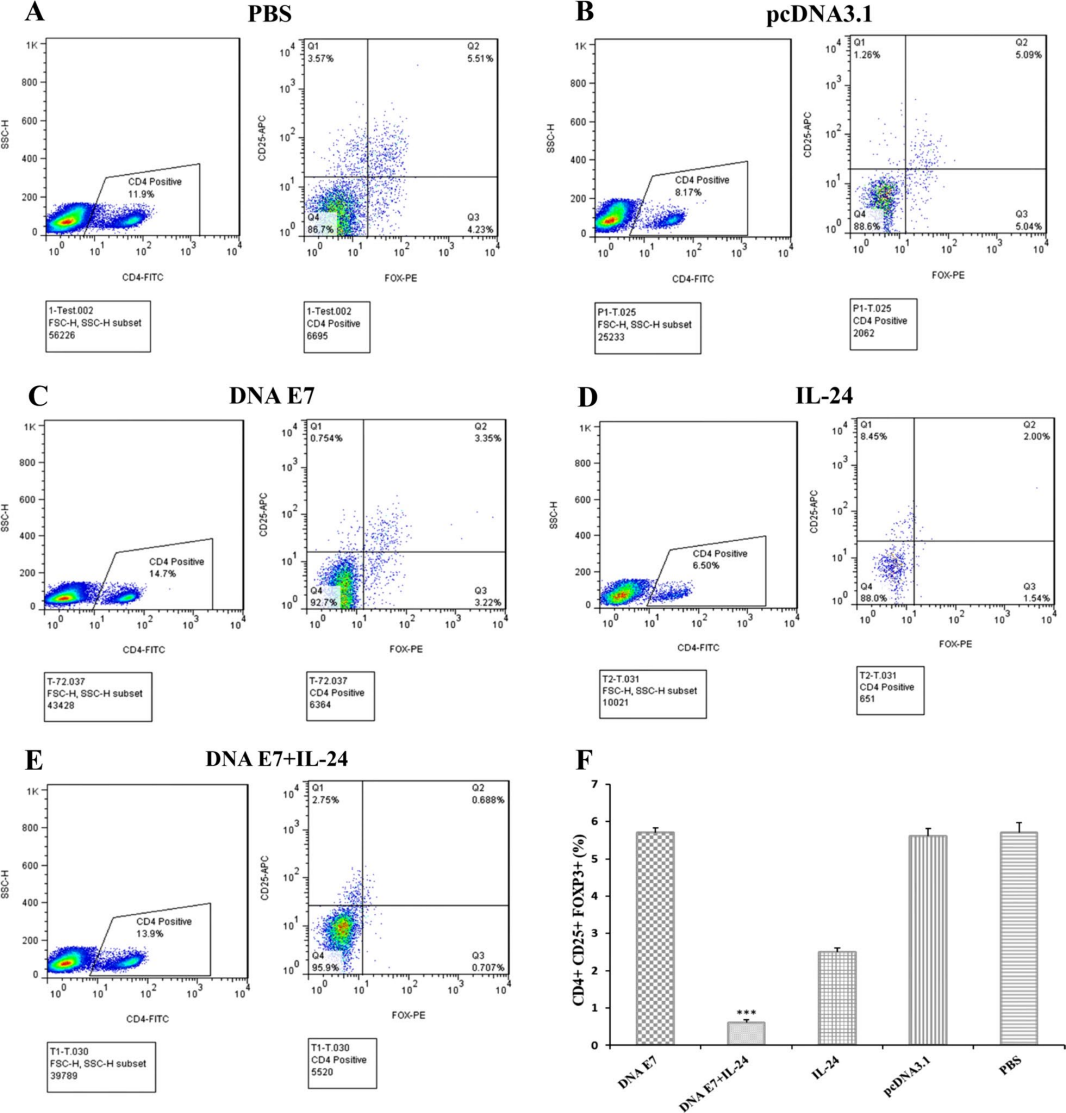
Characterization of depletion of CD4+ CD25+ Foxp3+ T cells in immunized mice. **A**–**E** Representative dot plots data demonstrating the frequency of CD4+ CD25+ Foxp3+  lymphocytes in the splenocytes of various immunized mice groups. Splenocytes were collected one week after last intratumoral injection of DNA vaccine and adjuvant, then subjected to flow cytometry analysis using the anti-CD4, anti-CD25, and anti-Foxp + antibodies. **F** Bar figures represent the percentages of CD4+ CD25+ Foxp3+ T cells (Treg) among CD4+ T cells from splenocytes. Mice treated with DNA E7 plus IL-24 had the lowest numbers of Treg cells from splenocytes among the other groups (*** p < 0.001). Data demonstrate mean ± one standard error of two independent experiments

Regulatory T cells are a specialized subpopulation of T cells with the ability to down-regulate the immune system [1]. On the other hand, Tregs are considered the key mediators of tumor progression, which is associated with their immune-suppressive nature [5]. The Treg population in the spleen of scarified mice was estimated using flow cytometry analysis. As displayed in Fig. 5, the percentage of regulatory T cell population was significantly higher (P < 0.001) in control groups compared to E7&IL-24 and E7 groups. In addition, the Treg population was reduced in the IL-24 group to 2% of count cells. It was also reported that the Treg population in the E7&IL-24 group was significantly lower than sole E7 (P < 0.001) or IL-24 (P < 0.01) groups. Therefore, IL-24 benefits from a potent capacity to suppress Tregs and subsequently boost antitumor immune responses.

### Intratumoral TRAIL and caspase-9 analysis

**Fig. 6:**
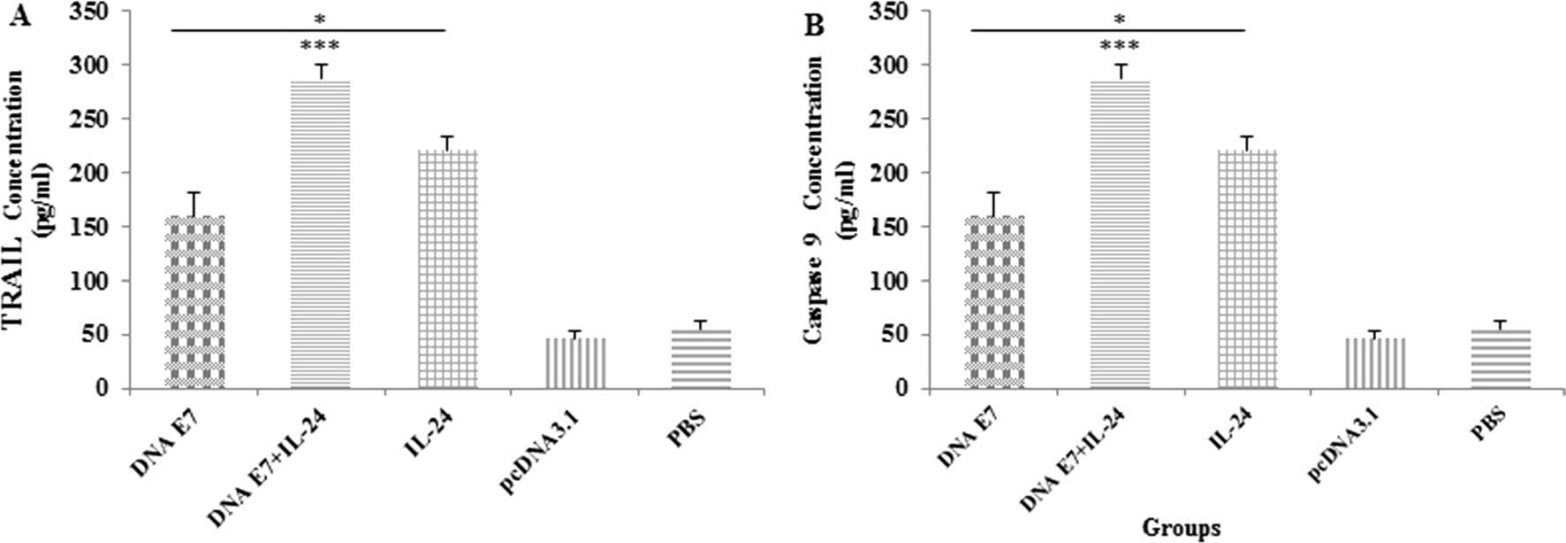
The levels of caspase-9 and TRAIL proteins in the tumor microenvironment. Mice were challenged with TC-1 tumor cells and immunized as described in the text. One week after immunization, mice groups were sacrificed and tumor tissue extracted and homogenized to form a uniform tumor lysate. The concentration of caspase-9 (**A**) and TRAIL (**B**) in tumor lysates determined by ELISA. Results are representative mean ± SD of three independent experiments. *P < 0.05; ***P < 0.001

It was demonstrated that tumor necrosis factor-related apoptosis-inducing ligand (TRAIL) mediates the apoptosis pathway in tumor cells without toxicity to the host [16]. Additionally, caspase-9 is a key player in the intrinsic apoptotic pathway, sparking apoptosis by initiating the caspase cascade-related signaling pathway [17]. TRAIL and caspase-9 in the tumor microenvironment were measured to investigate the mechanism through which our developed adjuvant vaccine destroys tumor cells. As predicted, the levels of both apoptotic factors were significantly higher (P < 0.001) among immunized groups compared to control groups. The TRAIL and caspase-9 in the mice treated with adjuvant E7&IL-24 vaccine were significantly higher (P < 0.001) in comparison with the E7 or IL-24 group. Moreover, although no significant difference was found between E7 and IL-24 groups in terms of the level of caspase-9, the concentration of TRAIL in the E7 group was lower (P < 0.05) than that of the IL-24 one (Fig. 6). In general, these data confirmed the governing role of apoptosis in destructing tumor cells and controlling the tumor progression by our proposed adjuvant vaccine.

### Immunohistochemical analysis

**Fig. 7:**
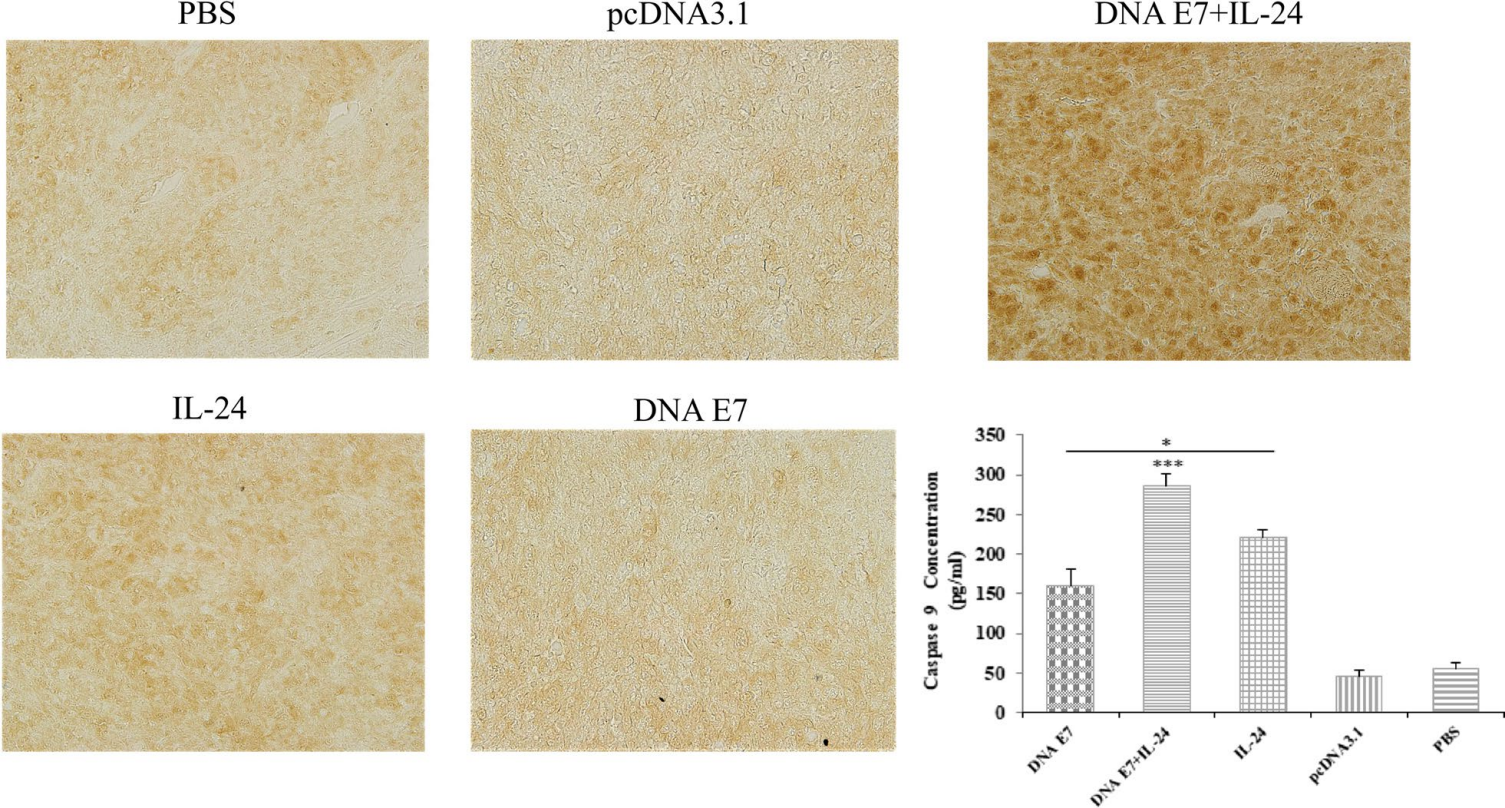
Immunohistochemical detection of IL-24 in tumor tissue of immunized mice. The tumor tissues were collected from mice sacrificed one week after the last immunization with DNA vaccine and expression of the IL-24 protein was determined by IHC (magnification: × 200). Stained areas of IL-24 expression were quantified using Image J software. The graph shows the average percentage (%) of the stained area in the tumor tissue section in different *groups. The results were recorded as the mean ± SD of three independent experiments. ***P < 0.001; *P < 0.05

The recruitment of IL-24 at the tumor site was validated and quantified by IHC. The formalin-fixed, paraffin-embedded tumor tissue was prepared, and stained tissues were visualized after treatment with the anti-IL-24 antibody (Fig. 7A). According to Fig. 7B, the infiltration of IL-24 at the tumor microenvironment of immunized groups was higher and the difference was statistically significant compared to control groups (P < 0.001). More importantly, the expression of IL-24 in the E7&IL-24 group was significantly higher (P < 0.001) in comparison with E7 and IL-24 groups. Furthermore, the obtained tumor tissue from the IL-24 group contained a higher amount (P < 0.05) of IL-24 cytokine when compared with the E7 group (Fig. 7B). The data suggest the synergistic effect of the E7 DNA vaccine on IL-24 engagement at the tumor site.

### Tumor size

**Fig. 8:**
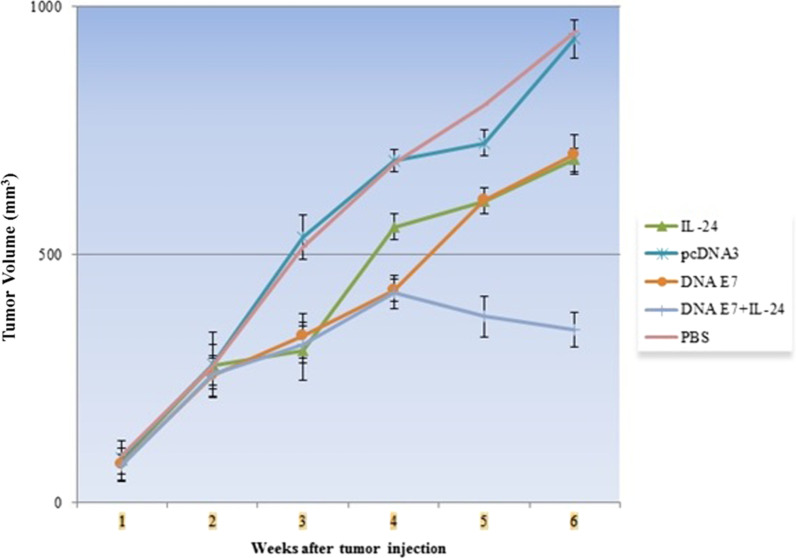
Efficacy of DNA vaccine and combination therapy in tumor cell (TC-1)-bearing mice. Tumor tissue was established through injection of TC-1 cells (1 × 10.^5^) subcutaneously. After twice immunization, tumor volume was calculated until 6th week post tumor challenge. The comparison of the difference between E7&IL-24 group and other groups (DNA E7, IL-24, pcDNA and PBS groups) was significant (p < 0.001). Data are represented as the mean ± SD (n = 6 mice per group). Significance for mouse survival was determined by Student’s t-test

This study focused on examining the effect of the E7 DNA vaccine, IL-24, and E7&IL-24 on the tumor progression in TC-1 tumor-bearing C57BL/6 mice. Six tumor-bearing mice were followed up every two days to measure the tumor size for six weeks. The mean of the tumor volume of mice is shown in Fig. 8. After the 3^rd^ week, the tumor volume of the study groups was substantially lower (P < 0.001) than that of the control groups. Measurements in the 4^th^ week revealed that although the difference between the tumor volume of E7&IL-24 and E7 groups was not noticeable, a smaller (P < 0.001) tumor was observed in both groups compared to the IL-24 group. From week 4 on, the tumor volume of the E7&IL-24 group followed a prominent regression trend so that the tumor size of this group on weeks 5 and 6 was considerably (P < 0.001) less than that of both E7 and IL-24 groups. Based on these results, IL-24 can efficiently enhance the tumor regression potential of the E7 DNA vaccine, especially in the long term.

### The effect of IL-10 blockade on IFN-γ, IL-10, and caspase-9 levels

**Fig. 9:**
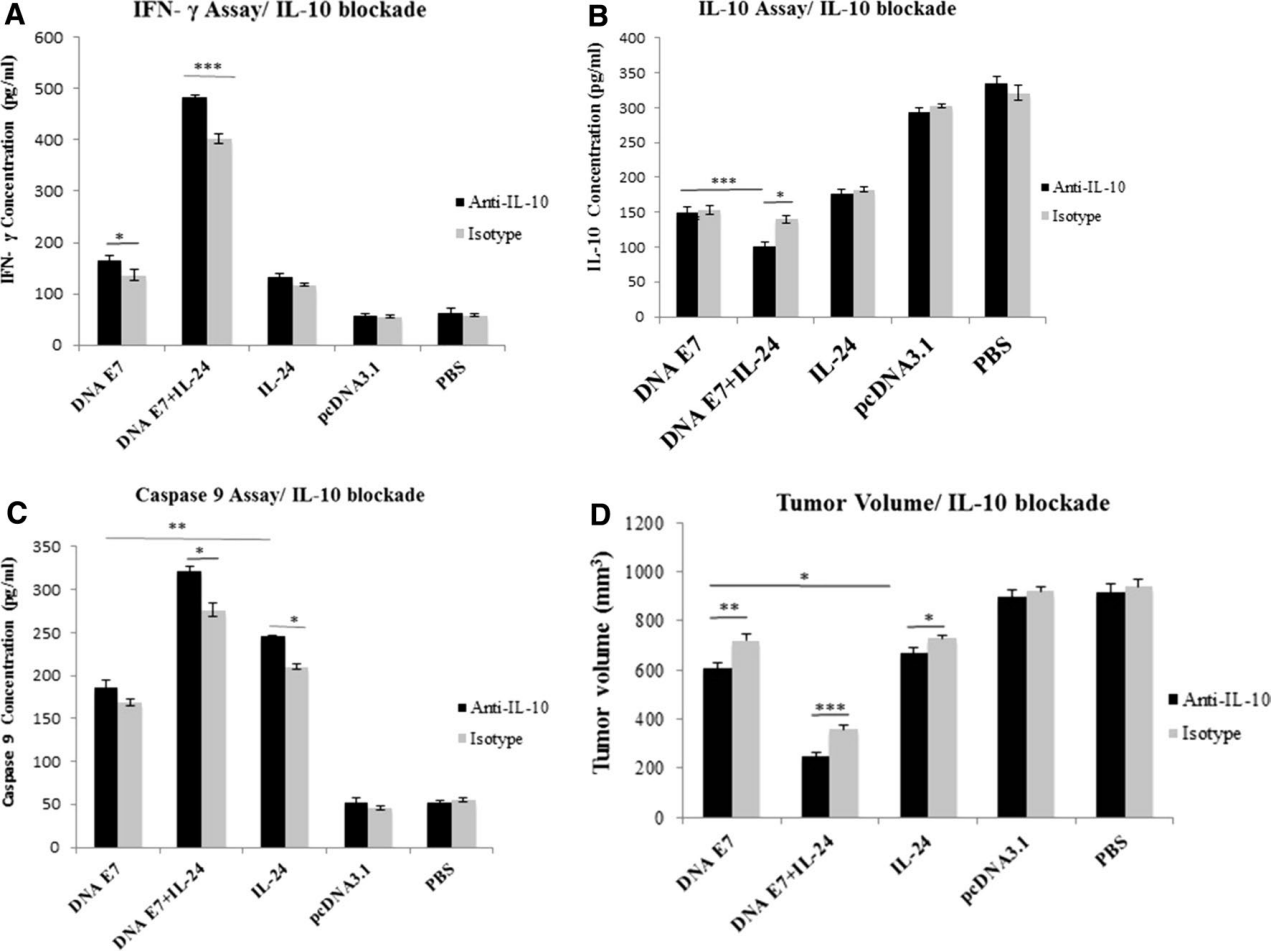
The impact of IL-10 blockade on anti-tumoral responses. **A** IFN-γ level in DNA E7&IL-24 (P < 0.001) and IL-24 (P < 0.05) groups highly increased when coupled with IL-10 blockade. **B** IL-10 level in DNA E7&IL-24 (P < 0.05) groups significantly decreased when coupled with IL-10 blockade. **C** The concentration of caspase-9 increased in DNA E7&IL-24 (P < 0.001) and IL-24 (P < 0.05) groups when treated with anti-IL-10 in comparison with isotype control. Also, **D** tumor volume regressed in DNA E7&IL-24 (P < 0.001), DNA E7 (P < 0.01) and IL-24 (P < 0.05) groups when treated with anti-IL-10 in comparison with isotype groups. These data are representative means ± SD of three independent experiments *P < 0.05; **P < 0.01; ***P < 0.001

To investigate whether or not, and to what extent, the suppression of the immunosuppressive IL-10 cytokine can enhance the outcome of our developed adjuvant vaccine, anti-IL-10 mAb was incorporated into the components of our immunization recipe. Then the levels of IFN-γ, IL-10 and caspase-9, and also the tumor size in mice receiving anti-IL-10-added vaccines were compared with those of mice treated with isotype homologs six weeks after tumor challenge. Regarding the level of IFN-γ, IL-10 blockade could induce higher levels of this cytokine in E7&IL-24 (P < 0.001) and E7 (P < 0.05) groups in comparison with isotype control groups (Fig. 9A). Measurement of IL-10 cytokine also showed that IL-10 blockade reduce the secretion of this cytokine in E7&IL-24 (P < 0.05) group in comparison with isotype control groups (Fig. 9B). Besides the lower levels (P < 0.001) of IL-10 within all immunized groups compared to control groups, a reduced amount (P < 0.001) of this cytokine was detected in the E7&IL-24 group in comparison with E7 group. These data underline the suppressive effect of IL-10 on the activation of immune cells (monocytes, macrophages, and the like), which mediate the production of IFN-γ.

Concerning the effect of IL-10 blockade on the expression of caspase-9, it was noted that the suppression of IL-10 augments (P < 0.05) the production of caspase-9 in E7&IL-24 and IL-24 groups when compared to their homologous isotype control groups, and no significant difference was observed in this respect in the E7 group (Fig. 9C). The IL-10 moderately hampers the programmed cell death pathway, suggesting its elimination to obtain the best result out of apoptosis-inducing therapeutic agents.

### The effect of IL-10 blockade on tumor status

The current study also examined the impact of IL-10 on tumor regression induced by E7&IL-24, E7, or IL-24 in parallel with our main study by following up the tumor volume for up to six weeks. Our results indicated the inefficient impact of IL-10 on the therapy outcomes regarding the tumor size. Smaller tumor size was detected in E7&IL-24 (P < 0.001), E7 (P < 0.01), and IL-24 (P < 0.05) groups when coupled with anti-IL-10 compared with isotype analogs (Fig. 9D). In sum, the incorporation of anti-IL-10 showed promising signs of complementary therapy in combination with cancer immunotherapy for more efficient tumor eradication.

## Discussion

The findings of this study indicated that immunization with E7 DNA vaccine adjuvanted with IL-24 could induce higher levels of immunogenicity. When we compare the E7 DNA vaccine adjuvanted with IL-24 to other groups (E7 DNA vaccine alone or control groups, namely, IL-24, pcDNA3, and PBS), we observed a higher lymphocyte proliferation level, CD8+ T cell activation, and higher Th1 cytokine levels (IFN-γ, TNF-α, and IL-10) and IL-4 levels, compared to other groups. To the best of our knowledge, it is the first study to evaluate IL-24 as an adjuvant for DNA vaccine development. Moreover, concerning the mechanism through which IL-24 assists tumor clearance, a rise in caspase-9 and TRAIL levels was detected, which indicates the high involvement of the apoptotic pathway. This result is compatible with that of a very recent study, confirming that IL-24 could elicit a higher number of apoptotic cells when added to the oncolytic vaccinia virus against colorectal cancer [18]. Eventually, the study further focused on assessing if dampening IL-10, as an immunosuppressive cytokine, has a positive effect on the antitumor responses of the adjuvanted vaccine. The results demonstrated an increase in the level of IFN-γ, which is relevant to the enhancement in the induction of CTL responses, along with an improvement in the reduction of tumor size concomitant with decreased Il-10 secretion and increased levels of caspase-9 as an apoptosis initiator protease.

Many DNA vaccine candidates have been evaluating pre-clinically or clinically against human papillomavirus type 16 (HPV16)- and 18 (HPV18)-caused cancers, especially cervical cancers. Among these studies. For example, among these studies, the VGX-3100 DNA vaccine, targeting E6 and E7 proteins of HPV-16 and HPV-18, was evaluated in a phase II clinical trial (NCT01304524) concerning its safety, efficiency, and immunogenicity in patients suffering from the cervical intraepithelial neoplasia grade 2/3 or 3. The side effects were limited to mild rash at the injection site or fatigue and nausea in some patients. Moreover, there was a significant rise in the population of activated CD8+ T cells and humoral responses among those receiving the vaccine rather than placebo-received ones, suggesting VGX-3100 as a promising DNA vaccine to thoroughly elicit adaptive immunity against HPV-driven cervical cancer [19]. Additionally, a more recent report has measured the 1.5-year lasting therapeutic effect of VGX-3100 after administration of the final dose and an efficacy index comparable to conventional surgery, introducing vaccination as a promising substitute for radical therapies [20].

The E7 protein of HPV, as one of the most prevalent tumor-associated antigens present at the surface of HPV-related malignant cells, is considered the ideal alternative for developing novel vaccines in the treatment of HPV-associated malignancies [21]. However, the E7 DNA vaccine administration alone has been reported to be poorly immunogenic with limited applications [22]. Several mechanisms can describe the immunogenic limitation of E7 DNA vaccines, including the evading mechanisms taken by the virus to escape the host recognition and tumor resistance mechanisms such as epitope alteration and deletion to escape T cell recognition, tolerance to antigens, and involvement of immunosuppressive cells such as Tregs and myeloid-derived suppressor cells [23, 24]. To circumvent this limitation and enhance the efficiency of DNA vaccines, a wide range of strategies have been implanted, including the optimization of codon usage [1], construction of recombinant DNA vaccines [25], and combination of DNA vaccines with other therapies (including chemotherapy), application of immune checkpoint blockade, and incorporation of cytokines and adjuvants [26–30].

Regarding the latter approach, the E7 DNA vaccine adjuvanted with toll-like receptors and natural killer cell ligands could efficiently increase IL-4 and IFN-γ in TC-1 tumor-bearing mice [26]. Further, employing synthetic poly-methyl methacrylate adjuvant with a recombinant E7 DNA vaccine could increase the level of TNF-α and IL-6 in cervical cancer models [31]. Consistent with those studies, the findings of the present study demonstrated significant increase in the level of IL-4, IFN-γ, and TNF-α in our proposed E7&IL-24 vaccine when compared to non-adjuvanted E7 vaccine or IL-24 alone.

Moreover, the capacity of some cytokines for addressing the low immunogenicity of DNA vaccines was assessed in previous research. Among these cytokine adjuvants, IL-2, IL-12, and GM-CSF are the most investigated [32–34]. IL-2 evaluation, as an adjuvant for an E7 DNA vaccine against HPV-16-related cervical cancer, suggested the potential of this immunoregulatory cytokine in higher infiltration, activation, and degranulation of CD8+ T cells, determined by an increase in IFN-γ and CD107a levels [35, 36]. More importantly, shifting the phenotype of E7-specific CD8+ T cells to T effector memory phenotype, which is necessary for antitumor protection and inhibiting tumor relapse, together with preventing the increase of splenic MDSC, as a marker of tumor progression, were among the other adjuvant effects of IL-2 on E7 DNA vaccine [36]. A similar survey on the adjuvant role of IL-12, as one of the most compelling immunostimulatory cytokines that prominently increased CTL responses and the level of IFN-γ, resulted in more efficient antitumor responses [37]. In another study, utilizing IL-12 in combination with the E7 DNA vaccine, delivered by chitosan nanoparticle, could enhance the secretion of IL-4 and IFN-γ whereas reducing the level of immunosuppressive IL-10 cytokine [38]. Similarly, in addition to an increase in IL-4 and IFN-γ levels, the current study results indicated that the combination of E7 and IL-24 could induce a decline in the level of IL-10.

Several studies have reported the potential of MDA7/IL-24 in inducing CTL proliferation [13], and apoptosis through the induction of mitochondrial apoptosis pathways and/or endoplasmic reticulum (ER) stress [39–41]. In one study, the antitumor effects of this cytokine against breast cancer were demonstrated to be applied through apoptosis and stress induction in ER, as well as the inhibition of cell growth and suppression of the self-renewal ability of cancer stem cells [42]. Our results regarding measuring the level of two apoptosis markers (i.e., TRAIL and caspase-9) highlighted the apoptotic pathway as the domineering mechanism behind the antitumor activity of IL-24. Similarly, the current study’s findings revealed that a combination of the E7 DNA vaccine with IL-24 significantly activates apoptotic pathways, and it was highly more than the results observed in separate E7 or IL-24 groups.

In addition to the investigations incorporating apoptotic antigens for developing DNA vaccines [43, 44], some studies attempted to increase the efficacy of such vaccines by modulating apoptotic pathways through the administration of pro-apoptotic adjuvants [45, 46]. In this regard, the incorporation of mutant pro-apoptotic caspases and influenza hemagglutinin DNA vaccine induced an augmentation in T helper (Th)2-associated immune responses (IL-4 and IgG1) and an enhancement in CD4+ T cell activation, highlighting the high adjuvant capacity of apoptosis-inducing reagents in developing DNA vaccines [47]. Likewise, the intradermal co-administration of a double antigen DNA vaccine with a plasmid encoding Bax, as a pro-apoptotic gene, resulted in a prominent rise in antibody and CTL responses, representative humoral immunity and effective cellular immunity, respectively [48]. In accordance with these results, our evaluation of the previously known pro-apoptotic properties of IL-24 (evident regarding the caspase-9 and TRAIL level promotion) and its capacity as an adjuvant for E7 DNA vaccine confirmed a significant enhancement in cellular immunity, indicated by increased IL-4, IFN-γ, and TNF-α cytokine production and CTL responses.

Furthermore, immunogenic cell death (ICD) plays a key role in prompting and regulating antitumor immune responses in the tumor microenvironment [49]. One of the most prominent functions of ICD is the ability to induce the upregulation of many damage-associated molecular patterns (DAMPs), subsequently leading to the activation of DCs, enhancing antigen presentation by these cells, and finally, provoking T cell death responses against malignant cells [50]. Many studies have focused on identifying, synthesizing, and introducing ICD-inducer agents due to their proven antitumor adjuvant properties and their potential in inducing highly durable protective immunity [51–53]. Based on our knowledge of ICD hallmarks [54] and immunological data, substantiating the stimulation of the adaptive immune system upon the incorporation of IL-24 adjuvant, in particular, the rise in lymphocyte proliferation and CTL responses together with modified cytokine levels, this hypothesis is strengthened that IL-24 may act as an ICD-inducer adjuvant to trigger antitumor immunity by means of DAMPs.

Investigations on the anti-tumor activity of MDA-7/IL-24 and its potential to modulate pro-immune Th1 responses in a murine syngenic model of fibrosarcoma highlighted IL-24 as a potent cytokine in inhibiting tumor growth and triggering the regression of tumor cells [55]. The efficacy of IL-24 as an adjuvant with oncolytic viruses was also evaluated. IL-24-expressing oncolytic vaccine virus, against lung cancer, destructed cancer cells through apoptosis and induced a decline in the level of STAT3 [56]. Based on the findings of the present study, although the administration of the E7 DNA vaccine or IL-24 alone was not effective in reducing the tumor volume, the adjuvant effect of IL-24 on the E7 DNA vaccine induced a significant reduction in the tumor volume and a high anti-tumor effect, in comparison to the administration of the vaccine alone, was observed, especially from week 4 on.

In another study, coupling vaccinia virus oncolytic virotherapy with IL-24 and GM-CSF gene therapy represented prominent cytotoxicity effects on cancerous cells with no noticeable detrimental impacts on healthy cells [18]. In line with these data and based on the mediating role of IL-24 in modulating systemic immunity against cancerous cells, a strong synergism was observed between the E7 DNA vaccine and IL-24 adjuvant in the context of lymphocytic proliferation and cytotoxic activity. It was further found that the co-administration of IL-24 with the E7 vaccine enhances the proliferation of E7-specific T cells. Compatible with our immunological data, a high synergism in eliciting the cytolytic action of cytotoxic T lymphocytes, along with tumor destructive and IFN-γ-releasing cells was detected in the adjuvant E7&IL-24 group when compared to IL-24 or even E7 group.

The abundance of Tregs exerts negative effects on immune responses, particularly on cellular immune responses. It was reported that IL-24 induces a positive anti-tumor effect by decreasing the activity of Tregs and increasing the activity of CD8+ T lymphocytes [57]. A high concentration of IL-24 could also downregulate the Treg percentage (i.e., FoxP3 mRNA) and promote CD4+ T cell proliferation in colorectal cancer [58]. In accordance with these results, the regulatory lymphocyte population in the spleen of mice in the co-administration (E7&IL-24) group considerably decreased in our study. This decline was also observed in the IL-24 group, which was significant compared to the E7 group alone.

As regards the role of IL-10 in cancer, it was demonstrated that this cytokine might act as a double-edged sword. In other words, although IL-10 is known as an anti-inflammatory or immunosuppressive cytokine [59], some studies revealed the immunostimulatory activity of this cytokine in several cancer types [60]. On the one hand, the presence of large amounts of IL-10 in the cervical tumor microenvironment was confirmed [61]; on the other hand, there was a negative correlation between the level of IL-10 and the expression of class I human leukocyte antigens (HLAs). It should be noted that a reduction in the level of class I HLAs facilitates the immune evasion of tumor cells [62]. Generally, it seems that in the case of cervical cancer, dampening IL-10 would help the proliferation of more CTLs in the tumor microenvironment and thereby enhancement of tumor regression [14]. In this study, it was observed that IL-10 blockade could increase the capacity of our adjuvant E7&IL-24 vaccine in eradicating tumor cells, which was further proved by an increase in the level of IFN-γ and caspase-9.

## Conclusion

Regarding the recent progresses in cancer immunobiology and immunotherapy, the administration of DNA vaccines is considered a cost-effective and efficacious method for cancer therapy. Considering the high incidence of cervical cancer linked to a high mortality rate in women worldwide, mass investigations were conducted to elucidate causative factors of this malignancy and to develop novel therapies against this disease. The data obtained in this study underline the potential of a recombinant E7 DNA vaccine containing MDA7/IL-24 as a genetic adjuvant in abating and reversing cervical cancer progression in vivo. Moreover, suppression of IL-10 cytokine by incorporating anti-IL-10 mAb improved the outcome of vaccination, which may be due to a decline in IL-10-mediated tumor invasion, as well as an increase in the proliferation of effective CTLs at the tumor site. The results suggest a potential candidate adjuvant vaccine against HPV-related cervical cancer with high immunogenicity driven by using the anti-tumor MDA-7/IL-24 cytokine.

## Supplementary Information

**Additional file 1. Fig. S1**: Detection of MDA/IL-24 expressions in vitro. Western blot analysis of the MDA/IL-24 expressions. Lane A = pcDNA 3.1, Lane 2 = MDA/IL-24 (~ 24 kDa). Lane3: ladder.

## Data Availability

Not applicable.

## Abbreviations

HPV: Human papillomavirus
Treg: Regulatory T cells
TLR: Toll-like receptor
IFN: Interferon
MDA-7: Melanoma differentiation associated gene-7
PBS: Phosphate buffer saline
CTL: Cytotoxic T lymphocyte
FBS: Fetal bovine serum
LDH: Lactate dehydrogenase
Treg: Regulatory T
TNF-α: Alpha tumor necrosis factor
TRAIL: Tumor necrosis factor-related apoptosis-inducing ligand
HPV16: Human papillomavirus type 16
HPV18: Human papillomavirus type 18
ER: Endoplasmic reticulum
HLAs: Human leukocyte antigens
