# Supplementary figures and images for: Enhanced synergistic antitumor effect of a DNA vaccine with anticancer cytokine, MDA-7/IL-24, and immune checkpoint blockade
Source: Virol J. 2022 Jun 25;19:106. doi: 10.1186/s12985-022-01842-x (PMC9233788; doi:10.1186/s12985-022-01842-x)

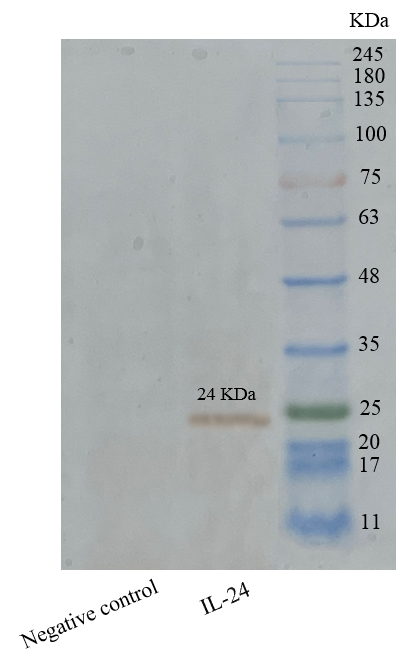

Supplement: Supplementary file 1 — Additional file 1. Fig. S1: Detection of MDA/IL-24 expressions in vitro. Western blot analysis of the MDA/IL-24 expressions. Lane A = pcDNA 3.1, Lane 2 = MDA/IL-24 (~ 24 kDa). Lane3: ladder. [file 12985_2022_1842_MOESM1_ESM.png]
